# Minimally Invasive Lumbar Pedicle Screw Fixation Using Cortical Bone Trajectory: Functional Outcomes

**DOI:** 10.7759/cureus.3462

**Published:** 2018-10-17

**Authors:** Yiren Chen, Sayantan Deb, Rashad Jabarkheel, Lan Pham, Mahesh Patel, Harminder Singh

**Affiliations:** 1 Neurosurgery, Stanford University Medical Center, Stanford, USA; 2 Neurosurgery, Stanford University School of Medicine, Stanford, USA; 3 Radiology, Santa Clara Valley Medical Center, San Jose, USA

**Keywords:** cortical screws, functional outcomes, fusion rates, oswestry, outcomes, retrospective review, roland-morris

## Abstract

Background

Pedicle screw fixation is currently the mainstay technique for lumbar spinal fusion; however, more minimally invasive techniques are available such as cortical screw fixation. Numerous studies have proven biomechanical equivalence or superiority for cortical screws but few studies have examined clinical outcomes in patients. Our study aims to examine functional outcomes, as well as fusion rates, in patients who underwent pedicle screw fixation using a cortical trajectory.

Methods

We retrospectively reviewed prospectively collected functional outcomes data on 10 patients with a degenerative lumbar disease who underwent cortical screw placement by the senior author. Oswestry Disability Index (ODI) and Roland Morris (RM) scoring were calculated preoperatively, at six to 12 weeks and at six to eight months. The Kruskal-Wallis test and Dunn’s multiple comparison were used to analyze differences in scores over time.

Results

We found that over time, cortical screw fixation resulted in a mean decrease of 27 from the baseline ODI at six to eight months (p = 0.014). Additionally, six out of seven (86%) patients who had at least 12 months of radiographic follow-up showed fusion.

Conclusions

Cortical screw fixation showed a decrease of 27 from the baseline ODI at six to eight months, which is comparable to changes from the baseline ODI reported in three, recent, large clinical trials examining functional outcomes following traditional pedicle screw fixation.

## Introduction

Pedicle screw fixation has been the mainstay technique for greater than 120,000 lumbar fusions performed in the United States every year [1]. However, the technique is invasive and requires significant lateral dissection to achieve the proper lateral to medial screw trajectory. This is particularly problematic in larger or overweight patients, resulting in longer incisions, extensive muscle dissection, and longer operative times. In recent years, a less invasive technique has been developed whereby screws are placed at the junction of the superior articular process and pars, angling more superiorly in a medial to lateral trajectory to achieve pedicle fixation. This technique is called cortical screw fixation.

There have been numerous studies examining the biomechanical properties of the cortical bone trajectory versus the traditional pedicle screw trajectory, overall showing the equivalence or superiority of cortical screws [2-12]. A study by Matsukawa et al. showed that cortical bone trajectory screws demonstrated a 26.4% greater mean pullout strength (p = 0.003) compared to pedicle screws. They also showed a 27.8% increased stiffness (p < 0.5) during cephalocaudal loading and 140.2% showed increased stiffness (p < 0.001) during mediolateral loading.

Despite the promising biomechanical studies, there is a dearth of literature on functional outcomes for patients that have undergone cortical screw fixation. Chen et al. recently published the first prospective cohort study on pain outcomes after cortical screws, showing that cortical screw patients had a trend towards less peak postoperative pain (p= 0.09) in the short term (one to three days post-surgery) but increased pain (p = 0.2) in the long term (six to eight months post-surgery) compared to pedicle screw patients [13]. In addition to this paper, there are only two other studies that look at clinical outcomes in cortical screw patients. Lee et al. showed, in a randomized non-inferiority trial in 79 patients, that six and 12-month fusion rates were similar, with similar pain amelioration and functional outcomes at follow-up [14]. In contrast, Sakaura et al. showed that cortical screw patients had a lower rate of fusion at 88.4% as compared to 96.3% in the pedicle screw group [15]. The goal of our present retrospective review is to assess the functional outcomes of patients at our institution who received cortical screw fixation for lumbar degenerative disease and evaluate the rate of fusion using this technique of spinal fixation.

## Materials and methods

Patient selection

This study is a retrospective review of prospectively collected data of patients who underwent cortical bone trajectory screw placement by the senior author at a large county hospital. Cortical bone trajectory pedicle screw placement is the standard of care for all patients at our institution operated on by the senior author with a lumbar degenerative disease requiring instrumented fusion of one to three levels. Patients with a pedicle diameter lower than seven millimeters on computed tomography (CT)/magnetic resonance imaging (MRI) receive instrumented fusion using traditional trajectory pedicle screw placement. Indications for fusion include unstable spondylolisthesis (instability on flexion-extension) and spondylolisthesis over grade two. An interbody graft is placed if there is a collapse of the disc space with resulting neuroforaminal stenosis.

A total of 10 patients were included in this study and underwent cortical trajectory screw placement. Informed consent was obtained from all patients prior to their procedure, which included consent for sharing intraoperative images and videos for teaching/publication purposes. All patients undergoing spinal surgery at Santa Clara Valley Medical Center (SCVMC) are encouraged to fill out functional outcome questionnaires before and after surgery in the clinic as the standard of care. The data is collected prospectively in real time in the patients’ electronic medical record. Institutional review board permission was obtained through SCVMC (#17-027) prior to analyzing any patient data, all of which was appropriately de-identified to comply with Health Insurance Portability and Accountability Act (HIPAA) protocols.

Surgical technique

The cortical screw technique requires significantly less muscle and lateral dissection, as well as a smaller longitudinal incision, as compared to traditional pedicle screw fixation. The starting point for cortical screws is at the junction of the superior articulating process and pars interarticularis, with a medial to lateral angulation (approximately 10 degrees lateral in the axial plane), and 25 degrees cranially in the sagittal plane. Actual angulations vary and were delineated by intraoperative fluoroscopy. A seven-millimeter minimum pedicle diameter cut-off was used in order to safely perform cortical screw fixation without lateral vertebral body breach or pedicle fracture. Arthrodesis was performed over the facet joints and in some cases of significant disc collapse, posterior lumbar interbody fixation (PLIF) using allograft was performed to restore disc height and decompress the neuroforamina.

Statistical methods

We examined functional outcomes in our cohort of patients at six to 12 weeks and six to eight months after cortical screw placement. Functional outcomes were measured by the Oswestry Disability Index (ODI) and Roland Morris (RM) disability questionnaire. The Kruskal-Wallis test and Dunn’s multiple comparison were used to analyze changes in disability measurements over time and at fixed intervals, respectively. A p-value of less than 0.05 was considered statistically significant. GraphPad Prism v 6.0 (GraphPad Software Inc., San Diego, California, US) was used to conduct all statistical analyses. Fusion was independently assessed by a neuroradiologist on anterior-posterior (AP) and lateral X-rays as well as flexion-extension films of the spine. Fusion was assessed by the presence of bridging bone between the intended levels of fusion, as well as stability on flexion-extension X-rays.

## Results

A total of 10 patients were included in the study and underwent cortical screw fixation. The mean age was 56 years, 40% were women, 50% smoked earlier, and the mean preoperative ODI was 70 while the mean preoperative RM was 15.3 (Table 1). Clinically, all patients presented with back pain and radicular pain. Flexion and extension films demonstrated spinal instability. Neuroforaminal stenosis, degenerative joint disease, and central stenosis were the predominant findings on MRI.

**Table 1:** Patient Characteristics SE: standard error; ODI: Oswestry Disability Index; RM: Roland Morris

Age (mean ± SE)	55.80 ± 2.30
Female (%)	40
Ever Smoker (%)	50
Preoperative ODI (mean ± SE)	70 ± 4.70
Preoperative RM (mean ± SE)	15.3 ± 1.37

On postoperative radiographic follow-up, one patient was lost to radiographic follow-up at six weeks due to insurance issues, making documentation of fusion unlikely. Six out of seven (86%) patients who had at least 12 months of radiographic follow-up showed fusion. Ultimately, seven out of 10 (70%) had documented radiographic fusion over a mean follow-up time of 14.7 months (Table 2). The presence or absence of an interbody did not affect fusion rates.

**Table 2:** Patient Presenting Symptoms, Pathology, and Postoperative Outcomes

Pathology	Presenting Pain Symptoms	Levels Fused	Interbody	Radiographic Fusion	Intra-operative Complications	Postoperative Complications
DJD and NFS at L4-5 and L5-S1	back pain and bilateral radicular leg pain	L4-S1	Yes	Partial, 12-month X-ray	None	Infection, small cerebrospinal fluid pooling near left L4 nerve root sleeve seen on reoperation
Central stenosis, left NFS - facet hypertrophy and synovial cyst	back pain and left radicular leg pain	L4-L5	No	Yes, 23-month X-Ray	None	None
DJD and NFS at L4-5 and L5-S1	back pain and bilateral radicular leg pain	L4-S1	Yes	Yes. 20-month X-ray	None	None
Central stenosis and left NFS L5-S1	back pain and neurogenic claudication	L5-S1	No	Yes, 24-month Flex-ex	None	None
DJD and left NFS	back pain and left radicular leg pain	L4-L5	No	Yes, 7-month X-ray	None	None
Central stenosis, left NFS	back pain and left radicular leg pain	L4-L5	No	No fusion mass. No instability on 7-month Flex-ex	Durotomy at L4-5, repaired primarily	None
DJD and central stenosis, L4-5 and L5-S1	back pain and right radicular leg pain	L4-S1	No	Yes, 16-month Flex-ex	Left L4 medial pedicle breach, no sequelae	None
Central stenosis, NFS at L3-4 and L4-5 synovial cyst	back pain and right radicular leg pain	L3-L5	L3-4 Yes, L4-5 No	Partial, 1.5-month X-ray	None	None
DJD and left NFS	back pain and bilateral radicular leg pain	L4-5	Yes	Yes, 17-month X-ray	None	None
Central stenosis, left NFS	back pain and left radicular leg pain	L4-5	Yes	Yes, 19-month X-ray	None	Superficial wound infection, local wound care
Key: DJD - degenerative joint disease, NFS - neuroforaminal stenosis, b/l - bilateral						

In terms of functional outcomes, eight patients reported disability measurements at all time intervals. Two patients failed to report disability measurements only at the six to 12 weeks' mark while another two patients failed to report disability measurements only at the six to eight months mark (Table 3). All patients reported some postoperative follow-up, either at six to 12 weeks or six to eight months.

**Table 3:** Cortical Screw Patient Series

Age	Pre-op ODI (%)	Pre-op RM	6-12 week ODI (%)	6-12 week RM	6-8 month ODI (%)	6-8 month RM	Ever Smoker	Significant Comorbidities
44	62	11	58	17	26	4	yes	COPD
59	50	10	24	6	30	9	yes	DM, OA, Sciatica
67	88	22	2	5	NA	NA	no	HTN, HLD
52	60	17	60	22	70	17	yes	HTN, HLD, DM
56	100	21	46	18	30	16	no	Lumbago
64	72	14	NA	NA	70	21	no	DM, HTN
58	66	18	64	20	54	20	yes	HTN
44	76	17	60	15	34	7	yes	HTN
55	68	11	52	5	NA	NA	no	None
59	58	12	NA	NA	30	8	no	DM, Migraine
Key: COPD – chronic obstructive pulmonary disease, DM – diabetes mellitus, HTN – hypertension, HLD – hyperlipidemia, OA – osteoarthritis, NA – Data not available								

**Figure 1:**
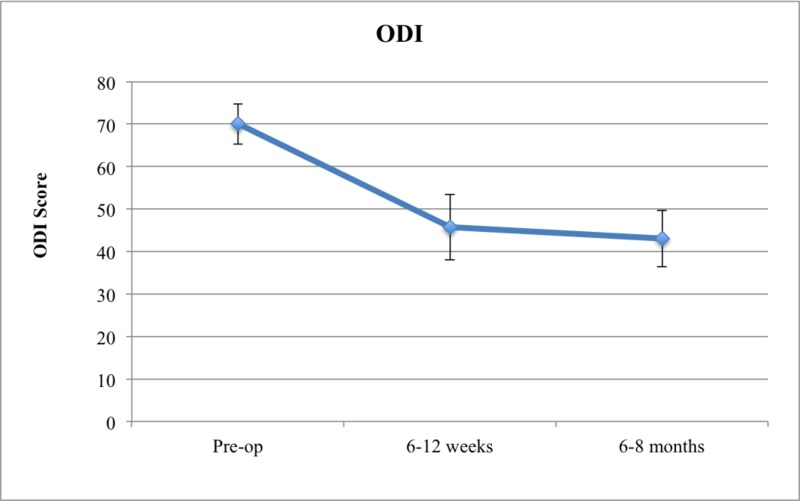
ODI Score in Cortical Screw Patients ODI: Oswestry Disability Index

**Figure 2:**
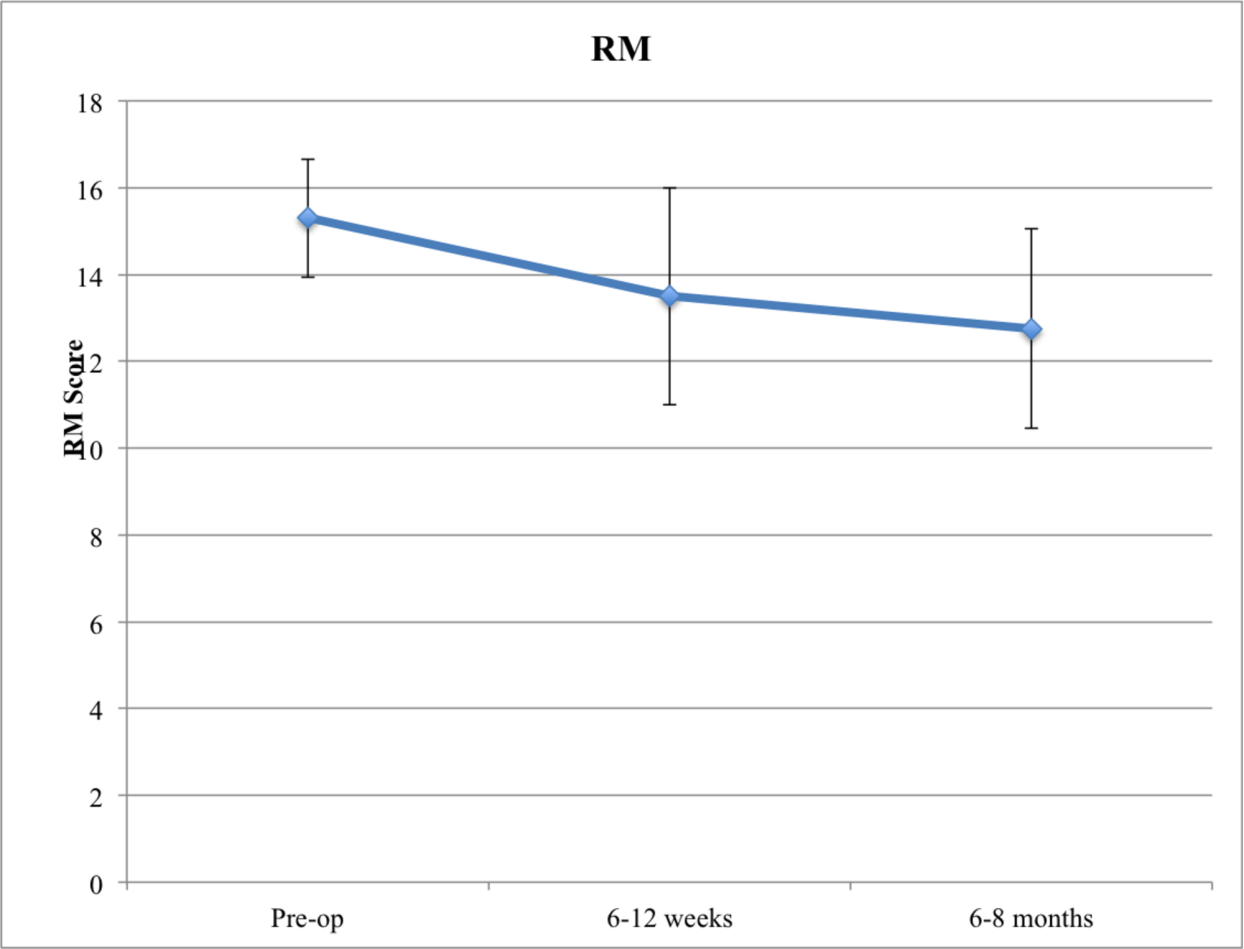
RM Score in Cortical Screw Patients RM: Roland Morris

Overall, it was found that patients who underwent cortical screw fixation had decreases both in their mean ODI and RM score over time (Figures 1-2).

In terms of ODI, the mean preoperative score was 70, decreasing to 45.75 at six to 12 weeks and 43 at six to eight months follow-up (Table 4). Using the Kruskal-Wallis test, it was found that patients who underwent cortical screw fixation had a statistically significant reduction of their ODI scores (p = 0.014). Additionally, using Dunn’s multiple comparison, it was found that the decrease in ODI scores from the preoperative to the six to 12 weeks mark and the decrease in ODI scores from preoperative to the six to eight months' mark were both significant (p = 0.04 and p = 0.03, respectively). The absolute reduction in ODI scores was most prominent between the preoperative and the six to 12 weeks' mark (ODI reduction = 24.25). Thus, most of the functional gains were made in the first six to 12 weeks after surgery.

**Table 4:** Long-Term Disability Measurement in Cortical Screw Patients SE: standard error; ODI: Oswestry Disability Index; RM: Roland Morris

	Pre-op	6-12 weeks	6-8 months	p-value (Kruskal-Wallis) over time within group	Dunn’s Multiple Comparisons
Mean ODI (mean ± SE)	70±4.70	45.75±7.69	43±6.62	0.014	Pre-op vs. 6-12 weeks: p=0.04
					Pre-op vs. 6-8 months: p=0.03
Mean RM (mean ± SE)	15.3±1.37	13.5±2.5	12.75±2.30	0.644	

In terms of RM, the mean preoperative score was 15.3, decreasing to 13.5 at six to 12 weeks and 12.75 at the six to eight months follow-up. The down-trending RM scores did not achieve a statistical significance (p = 0.644).

## Discussion

**Figure 3:**
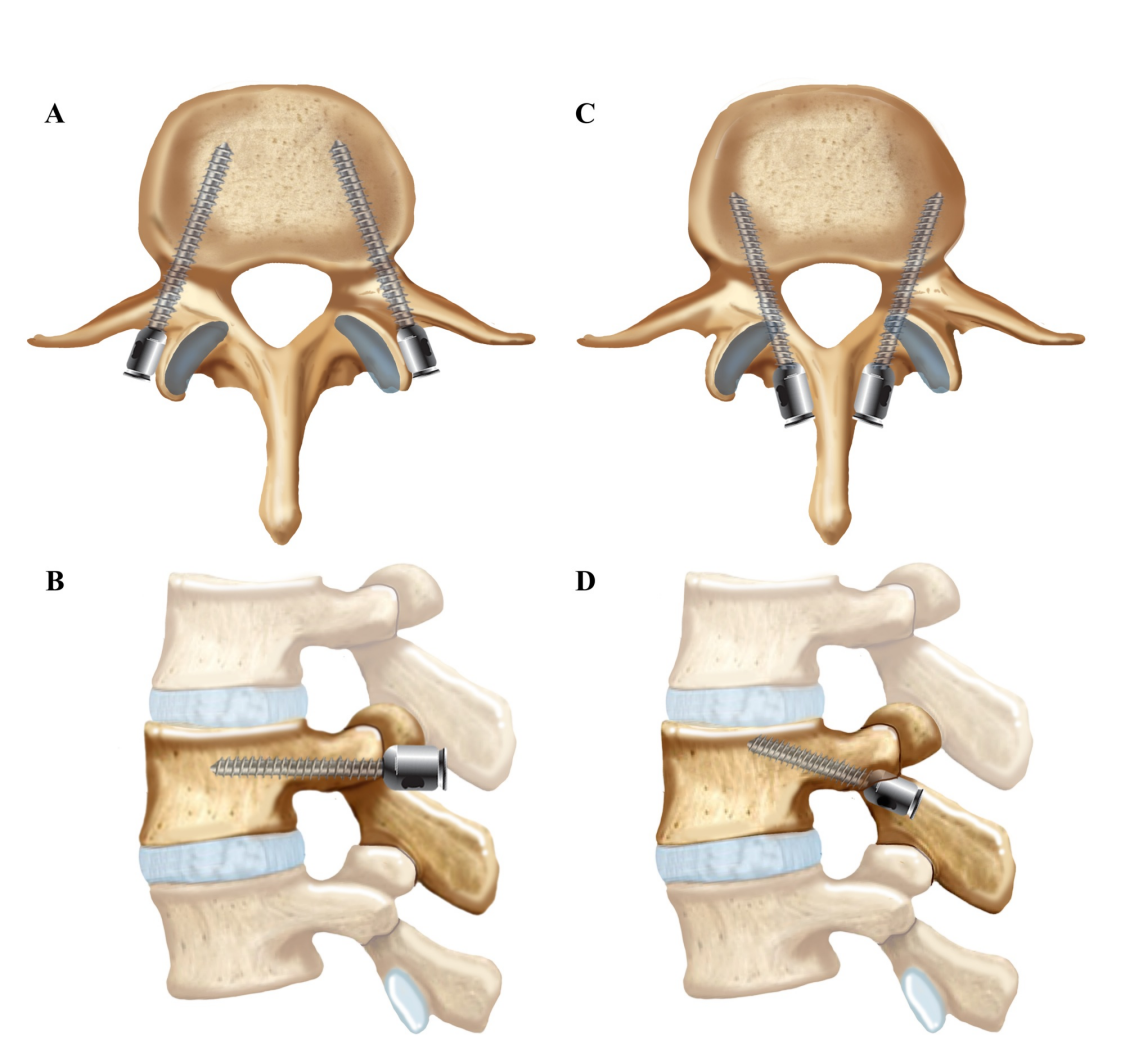
Traditional Pedicle versus Cortical Screw Fixation Trajectories (A and B) Traditional pedicle screw trajectories in the (A) axial and (B) sagittal views (C and D) Cortical screw trajectories in the (C) axial and (D) sagittal views

**Figure 4:**
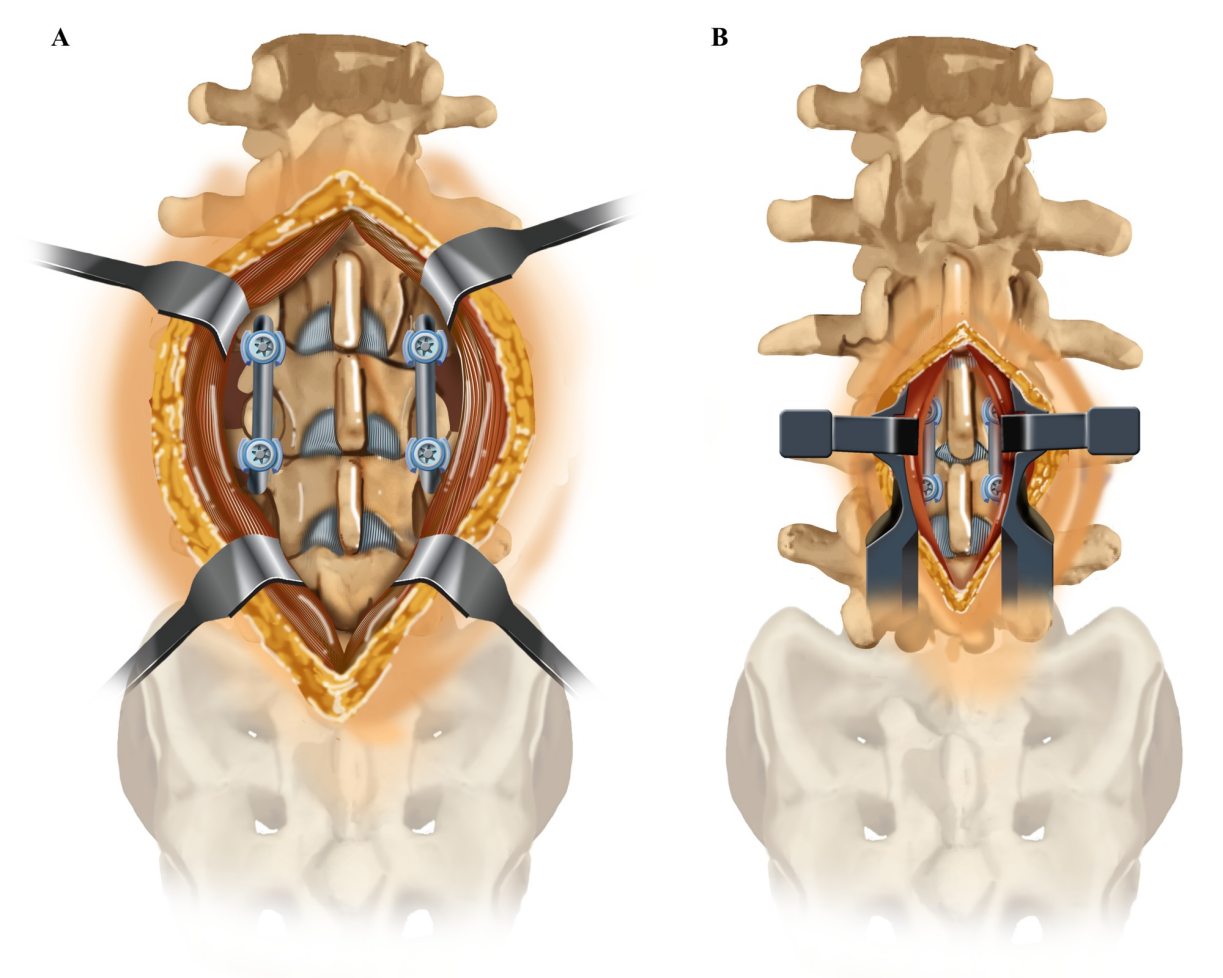
Soft Tissue Exposure Required for One Level Lumbar Fixation (A) Traditional pedicle fixation (B) Cortical screw fixation

Cortical screws are a minimally invasive alternative to pedicle screw fixation (Figures 3-4). Several biomechanical studies have shown the cortical bone trajectory to be equivalent, if not superior, to the traditional technique for pedicle screw fixation [2-12]. However, few studies have compared clinical and functional outcomes between cortical bone trajectory screw fixation and traditional pedicle screw fixation. In our study, we evaluated the functional outcomes associated with cortical screw fixation by measuring the ODI and RM scores of patients six to eight months after surgery. Additionally, we assessed the fusion rate after cortical screw fixation by having an independent neuroradiologist assess anteroposterior (AP) and lateral X-rays as well as flexion-extension films of patient’s spines.

We found that cortical screw fixation results in a statistically significant functional improvement for patients with a lumbar degenerative disease and spinal instability as measured by ODI. Specifically, we found that, on average patients who underwent cortical screw fixation experienced a decrease of 27 from the baseline ODI at six to eight months follow-up. The majority of the functional improvement was seen by the six to 12 weeks follow-up, as patients reported a change in the baseline ODI of -24.25 by that mark. This early improvement shows that approach-related discomfort from this less invasive technique resolves early and that the rigid spinal fixation accorded by cortical trajectory screws is very effective at immobilizing the spine and reducing the pain stemming from spinal instability.

Although our present study reported progressive decreases in RM scores with cortical screw fixation, the average decrease was not found to be statistically significant. In general, while RM and ODI scores are the two predominant scales used to assess functional disability from back pain, it has been noted by Roland et al. that ODI scores are better for assessing functional outcomes in patient populations with a chronic severe disability like ours [16]. Overall, the ODI is considered by many as the gold standard for measuring the degree of disability and estimating the quality of life in a person with low back pain, as it more fully captures the psychological and social problems associated with chronic low back pain [17].

From our present study, it can be concluded that cortical bone trajectory screw fixation results in improved functional outcomes for patients. However, the question remains as to how these functional outcomes following cortical screw fixation compare to those following traditional pedicle screw fixation. Several, recent, large-scale trials examining functional outcomes following traditional pedicle screw fixation provide a source of comparison for our data. Specifically, Ghogawala et al. found that traditional pedicle screw fixation results in a decrease of 12.4 from the baseline ODI at six weeks and of 25.9 at six months [18]. Forsth et al. found that traditional pedicle screw fixation results in an average decrease of 14 or 16 from the baseline ODI at two years depending upon whether the patient population had an absence or presence of degenerative spondylolisthesis, respectively [19]. Additionally, Lee et al. found that pedicle screw fixation with PLIF resulted in an average decrease of 19.2 from the baseline ODI at three months, 25.8 at six months, and 25.5 at one year. A quick comparison of our data (which showed a decrease of 25 from the baseline ODI at six to 12 weeks, and 27 at six to eight months) with results from the above studies demonstrates that functional outcomes following cortical screw fixation are similar to those reported for traditional pedicle screw fixation. Notably, our patient’s had similar decreases in ODI in comparison to Lee et al.’s cohort, all of whom received supplementary PLIF, despite less than half of our cohort receiving PLIF. We reserved PLIF only for cases where there was severe disk collapse or grade two spondylolisthesis at any spinal segment. There was no statistically significant difference in ODI scores between patients who received PLIF and those who did not in our series. The presence or absence of an interbody did not affect the fusion rate in our patients.

Traditional pedicle screw fusion rates have been cited as high as 96.3% by Sakura et al. and were found to be 89.5% with PLIF by Lee et al. Our fusion rates were 86% (six out of seven) in patients who had at least one year of radiographic follow-up. Our overall fusion rate was 70% (seven out of 10) with a mean follow-up of 14.7 months. Despite their lower fusion rate, our patients had excellent functional outcomes at six to eight months after surgery. This suggests that even in the absence of radiographic fusion, cortical screws are more than adequately immobilizing a joint, resulting in less pain and thus better outcomes. Further support for this notion comes from the fact that even in the case of our three patients that did not show radiographic fusion (two partial, one no fusion), none showed signs of instability on imaging as defined by movement on flexion-extension, haloing around screws, and screw pullout or fracture.

There are some limitations to our study, including the lack of randomization and the small sample size. This is also a single-institution study, with all surgeries performed by a single surgeon only. Future randomized studies with a greater number of patients and longer follow-up will be needed to firmly establish cortical trajectory pedicle fixation as a first-line option for lumbar spinal fixation in degenerative spine disease.

## Conclusions

This is the first study to show a significant functional improvement in degenerative joint disease patients with spinal instability following cortical screw fixation, with or without an accompanying PLIF. The functional improvement seen was equivalent to the functional improvement seen in patients with traditional lumbar pedicle fixation in recently published large series. Future randomized studies with a greater number of patients and longer follow-up will be needed to firmly establish a cortical trajectory pedicle fixation as a first-line option for lumbar spinal fixation in degenerative spine disease and spinal instability.
